# Revealing Cavin-2 Gene Function in Lung Based on Multi-Omics Data Analysis Method

**DOI:** 10.3389/fcell.2021.827108

**Published:** 2022-01-31

**Authors:** Changsheng Li, Jingyu Huang, Hexiao Tang, Bing Liu, Xuefeng Zhou

**Affiliations:** ^1^ Department of Thoracic Surgery, Zhongnan Hospital of Wuhan University, Wuhan, China; ^2^ Department of Pulmonary and Critical Care Medicine, Zhongnan Hospital of Wuhan University, Wuhan, China; ^3^ Wuhan Research Center for Infectious Diseases and Cancer, Chinese Academy of Medical Sciences, Wuhan, China

**Keywords:** relevance vector machine, adaboost, lung cancer, pulmonary injury, cavin-2

## Abstract

Research points out that it is particularly important to comprehensively evaluate immune microenvironmental indicators and gene mutation characteristics to select the best treatment plan. Therefore, exploring the relevant genes of pulmonary injury is an important basis for the improvement of survival. In recent years, with the massive production of omics data, a large number of computational methods have been applied in the field of biomedicine. Most of these computational methods are devel-oped for a certain type of diseases or whole diseases. Algorithms that specifically identify genes associated with pulmonary injury have not yet been developed. To fill this gap, we developed a novel method, named AdaRVM, to identify pulmonary injury-related genes in large scale. AdaRVM is the fusion of Adaboost and Relevance Vector Machine (RVM) to achieve fast and high-precision pattern recognition of pulmonary injury genetic mechanism. AdaRVM found that Cavin-2 gene has strong potential to be related to pulmonary injury. As we known, the formation and function of Caveolae are mediated by two family proteins: Caveolin and Cavin. Many studies have explored the role of Caveolin proteins, but people still knew little about Cavin family members. To verify our method and reveal the functions of cavin-2, we integrated six genome-wide association studies (GWAS) data related to lung function traits, four expression Quantitative Trait Loci (eQTL) data, and one methylation Quantitative Trait Loci (mQTL) data by Summary data level Mendelian Randomization (SMR). We found strong relationship between cavin-2 and canonical signaling pathways ERK1/2, AKT, and STAT3 which are all known to be related to lung injury.

## Introduction

The decline of lung function could predict mortality. Several studies have found that the decline of lung function is closely related to the occurrence of many diseases such as cardiovascular disease, lung cancer, and non-respiratory cancer (Duprez and Jacobs, 2018). Moreover, the measurement of lung function changes is the key to the diagnosis of many lung diseases such as Chronic Obstructive Pulmonary Disease (COPD) and Cystic Fibrosis (CF) (Shrine et al., 2019). These lung diseases especially COPD and CF are one of the causes of significant morbidity, mortality worldwide. For example, according to a study by (Ding et al., 2019), COPD is the fourth leading cause of death on Earth. In the diagnosis of these pulmonary diseases, the measurement of lung function indicators is the main basis to judge the changes in the disease condition and degree. Therefore, it is necessary to understand the regulatory mechanism behind lung function to prevent and treat lung function decline.

The regulation of lung function is complicated and has not been revealed yet. Governing lung function is mediated by a variety of cells such as complex endothelial and epithelial cells, dendritic cells, alveolar macrophages, and fibroblasts (Suzuki et al., 2008). They work together to deal with lung injury by triggering inflammation and an immune response. Caveolae are commonly found in lung epithelial cells, endothelial cells, adipocytes and fibroblasts (Murata et al., 2007). Caveolae are flask—shaped invaginated structures on the plasma membrane of mammalian cells, and are involved in many important cellular functions, such as endocytosis and endocytosis transport, and cell signal transduction (Parton et al., 2018). Also, this structure has been proved to play an important role in regulating various lung diseases such as asthma, COPD, CF, and acute lung injury. The structure and function of Caveolae are mediated by Caveolins family proteins and Cavins family proteins (Parton et al., 2020). These proteins are highly expressed in a variety of lung cells. To sum up, further research of these proteins is helpful to reveal the mechanism of lung function injury.

Cavin family proteins include Cavin-1, Cavin-2, Cavin-3, and Cavin-4, which govern the expression and morphology of Caveolae. Cavin-2 protein is mainly responsible for controlling the change of the Caveolae shape. For instance, the over-expression of Cavin-2 could cause the deeper invagination of Caveolae. Cavin2, also known as Serum Deprivation Protein Response (SDPR) protein was described as a protein induced during serum deprivation (Chettimada et al., 2015). Subsequently researchers undertook an in-depth study of Cavin-2 and found that this protein is essential for cell proliferation, migration and invasion. SDPR has been reported as a tumor suppressor in a variety of cancers, such as breast, liver, stomach, and endometrial cancers. Studies have shown that SDPR works in cancers because of the SDPR gene inactivation by methylation (Ozturk et al., 2016). Additionally, the absence of Cavin-2 was found to result in the loss of Caveolae of endothelial cells in lung tissues (Hansen et al., 2013). However, little information was available on the role of Cavin-2 in the lung function injury.

Thousands of SNPs associated with complex traits have been identified through GWAS. Most of these identified loci are located in intergenic regions and how they affect phenotypes through genes or pathways is difficult to elucidate (Watanabe et al., 2017). One possible explanation is that these susceptible sites alter complex traits in individuals by regulating methylation levels of a gene or inhibiting its expression. As eQTL refers to some variation sites on chromosomes that specifically regulate mRNA and protein expression levels of a gene, some of the variants overlap with SNPs found by GWAS, suggesting that they may be involved in the regulation of gene expression (Li et al., 2015). Moreover, mQTL was applied to recognize SNP sites significantly correlated with the methylation level of a gene (McRae et al., 2018). DNA methylation usually acts as an inhibitor of gene transcription. It is clear that both mQTL and eQTL can change their expression patterns by mutating at a single locus. Therefore, the association between Cavin-2 gene expression and lung function injury can be found through the conjoint analysis of GWAS and two different types of QTL data.

Although the previous studies have found multiple genes that are related to pulmonary injury, the speed of revealing the disease-causing genes of pulmonary injury still does not meet the needs of treatment. With the continuous maturity of sequencing technology, multi omics data showed a blowout growth. This provides support for the application of computing methods in biomedical field (Zhao et al., 2020a; Zhao et al., 2021a). Computational methods have been applied to the association and interaction between mutation sites (Peng and Zhao, 2020), genes (McKinney et al., 2006; Zhao et al., 2020b), various RNAs (Asefpour Vakilian, 2020), proteins (Zhao et al., 2020c; Noé et al., 2020), metabolites (Zhao et al., 2020d), drugs (Tianyi et al., 2020) and diseases, and various databases have emerged (Zhao et al., 2021b). It has become a common method to mine disease pathogenesis on a large scale by integrating a variety of different data for modeling and analysis. However, most of these methods focus on one kind of disease or all diseases. Each disease has its own specificity, which makes us need to design algorithms to identify its related genes for the study of pulmonary injury. In this paper, we fused Adaboost with Relevance Vector Machine (RVM) to design a pipeline for the identification of pulmonary injury-related genes.

## Methods

### Workflow

Firstly, we obtained 193 genes which are reported to be related to pulmonary injury in DisGeNET (Piñero et al., 2020). Then, we extracted the gene interaction information from String database (Szklarczyk et al., 2021). We extracted expression information of these genes from biogps database (Wu et al., 2016). Then, we did feature combination for each gene. Finally, we identified pulmonary injury-related genes by AdaRVM.

**FIGURE 1:**
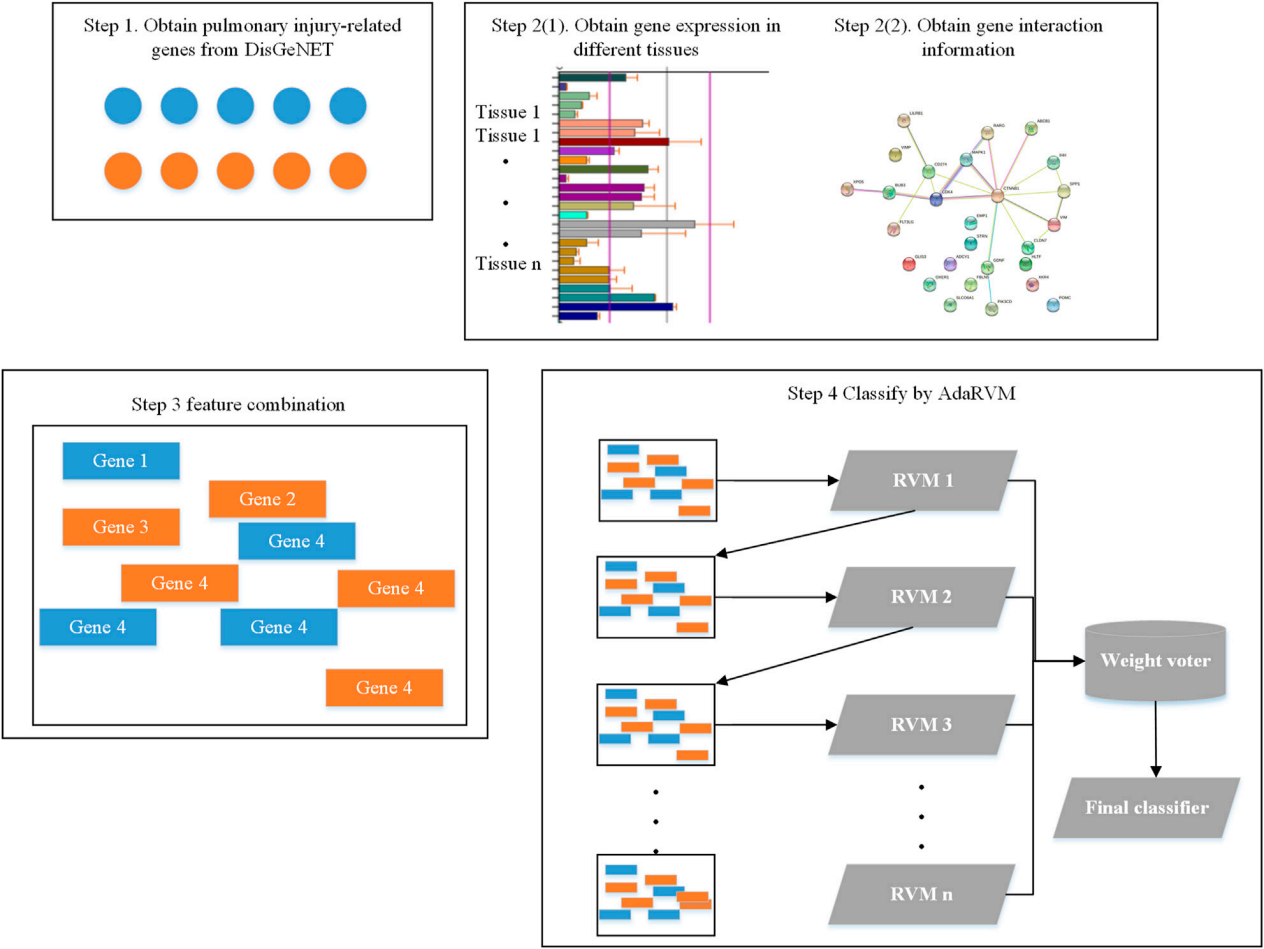
Workflow of identifying pulmonary injury-related genes.

The whole workflow is shown in Figure 1.

### Feature Combination

We obtained the expression of all genes in different tissues from Biogps database. We totally obtained gene expression in 13 tissues. For each gene, the feature of it would be:

$$Gene_{1}=\left[E_{1},E_{2},...,E_{13}\right]$$ (1)

$E_{i}$ represents the expression of gene in *i*th tissue.

Then, we obtained gene interaction information from string database. We obtained 193 pulmonary injury-related genes in DisGeNET and found 1,019 genes can interact with at least one of them. The interactions between them could be the second feature of genes.

$$Gene_{1}=\left[G_{1},G_{2},...,G_{1212}\right]$$ (2)

$G_{i}$ represents whether *i*th gene can interact with gene 1. If gene1 can interact with *i*th gene, $G_{i}$ = 1, otherwise, $G_{i}$ = 0.

Since the dimension of interaction feature for each gene is very high, we used Principal components analysis (PCA) to reduce the dimension. We put all genes together and used PCA to remain the 99% information. After PCA, the dimension of interaction feature for each gene is 237.

Finally, we combined interaction feature and expression feature together to get the final gene feature.

### RVM

The RVM model can give the conditional probability distribution of the target variable t given an input vector x:

$$p\left(t|x,w,\beta \right)=N\left(t|y\left(x\right),\beta^{-1}\right)$$ (3)

$\beta =\sigma^{-2}$ is the noise accuracy.

The mean value is given by the linear model:

$$y\left(x\right)=\sum_{i=1}^{M}w_{i}\varphi_{i}\left(x\right)=w^{T}\varphi \left(x\right)$$ (4)

The basis function is given by the kernel function. Each data point in the training set is associated with a kernel function. The general expression is:

$$y\left(x\right)=\sum_{n=1}^{N}w_{n}k\left(x,x_{n}\right)+b$$ (5)

The formula here is similar to the predictive model formula in support vector regression.

Suppose the N observation data of the input vector x are aggregated into a data matrix X , the n th row is $x_{n}^{T}$ , the objective value is $t={\left(t_{1},...,t_{N}\right)}^{T}$ , the likelihood function is:

$$p\left(t|X,w,\beta \right)=\prod_{n=1}^{N}p\left(t_{n}|x_{n},w,\beta \right)$$ (6)

The prior distribution on the parameter vector w is introduced, and the Gaussian prior of zero mean is considered. However, the key difference of RVM is that a separate super parameter $w_{i}$ is introduced for each weight parameter $a_{i}$ , rather than a shared super parameter. The prior form of weight is:

$$p\left(w|a\right)=\prod_{i=1}^{M}N\left(w_{i}|0,a_{i}^{-1}\right)$$ (7)

$a_{i}$ represents the accuracy of the corresponding parameter $w_{i}$ .

When the model evidence for these hyperparameters is maximized, most of them tend to infinity, and the posterior probability distribution of the corresponding weight parameters is concentrated near 0. Then the associated basis function will have no effect on the prediction of the model, so it can be removed to form sparsity.

The posterior probability distribution of weights is still Gaussian, in the form:

p(w|t,X,a,β)=N(w|m,∑) (8)

where $m=\beta \sum \varphi^{T}t,\sum ={\left(aI+\beta \varphi^{T}\varphi \right)}^{-1}$ .

After convergence, it can be found that part of the hyperparameter $a_{i}$ values tend to infinity, and the weight parameter $w_{i}$ corresponding to these hyperparameters has a posterior probability distribution with a mean and variance of 0. Then the corresponding parameters and basis function $\varphi_{i}\left(x\right)$ are erased from the model.

### AdaRVM

Adaboost generally uses a single-layer decision tree as its weak classifier. The single-layer decision tree is the most simplified version of the decision tree. It has only one decision point. That is to say, if the training data has multi-dimensional features, the single-layer decision tree can only select one of the one-dimensional features to make decisions. In our method, AdaRVM uses RVM as a weak classifier.

There are four steps to achieve Adaboost. Suppose our training set sample is:

$$T=\left\{\left({\text{x}}_{1}{\text{,y}}_{1}\right)\text{,}\left({\text{x}}_{2}{\text{,y}}_{2}\right)\text{,}...\text{,}\left({\text{x}}_{m}{\text{,y}}_{m}\right)\right\}$$ (9)

The output weight of the *k*th weak learner of the training set is:

$$D\left(k\right)=\left({\text{w}}_{k1}{\text{,w}}_{k2}\text{,}...{\text{,w}}_{km}\right){\text{w}}_{1i}=\frac{1}{m}$$ (10)

Where m is the number of training samples.

The first step is to get the weighted error rate of the *k*th weak classifier $G_{k}\left(x\right)$ on the training set.

$$e_{k}=p\left(G_{k}\left(x_{i}\right)\neq y_{i}\right)=\sum_{i=1}^{m}w_{ki}I\left(G_{k}\left(x_{i}\right)\neq y_{i}\right)$$ (11)

The second step is to obtain the weight coefficient of the weak learner. For the binary classification problem, the weight coefficient of the *k*th weak classifier $G_{k}\left(x\right)$ is

$$a_{k}=\frac{1}{2}\log\frac{1-e_{k}}{e_{k}}$$ (12)

It can be seen from the above formula that if the classification error rate $e_{k}$ is larger, the corresponding weak classifier weight coefficient $a_{k}$ is smaller. In other words, a weak classifier with a small error rate has a larger weight coefficient.

The third step is to update the sample weight D. Assuming that the weight coefficient of the sample set of the *k*th weak classifier is $D(k)={(\text{w}}_{k1}{\text{,w}}_{k2}\text{,}...{\text{,w}}_{km}{)\text{ w}}_{1i}=\frac{1}{m}$ , the weight coefficient of the sample set of the corresponding k+1 weak classifier is:

$${\text{w}}_{k+1,i}=\frac{{\text{w}}_{ki}}{Z_{K}}\exp\left(-a_{k}{\text{y}}_{i}{\text{G}}_{k}\left({\text{x}}_{i}\right)\right)$$ (13)

Where $Z_{K}$ is normalization factor

$${\text{Z}}_{k}=\sum_{i=1}^{m}{\text{w}}_{ki}\exp\left(-a_{k}{\text{y}}_{i}{\text{G}}_{k}\left({\text{x}}_{i}\right)\right)$$ (14)

The last step is weighted voting, and the final strong classifier is:

$$f\left(x\right)=gn\left(\sum_{k=1}^{K}a_{k}{\text{G}}_{k}\left(\text{x}\right)\right)$$ (15)

## Results

### Method Evaluation

**FIGURE 2:**
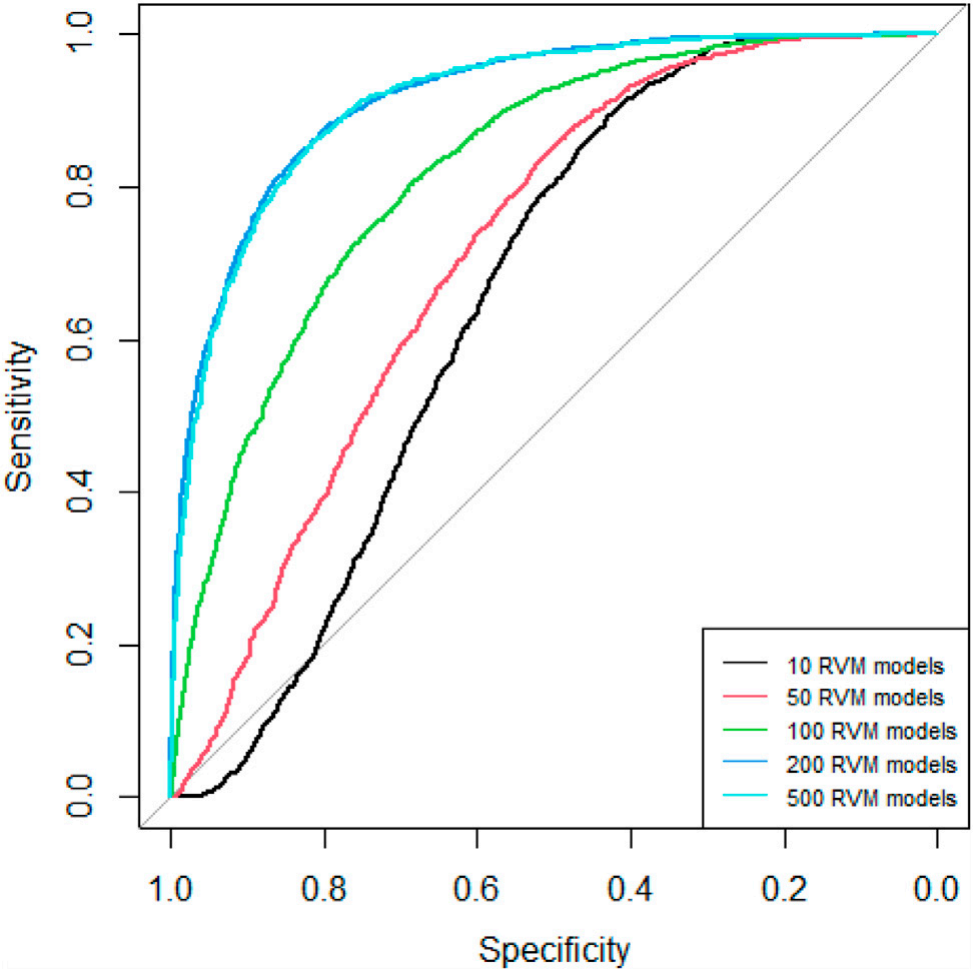
ROC curves of different RVM models.

**FIGURE 3:**
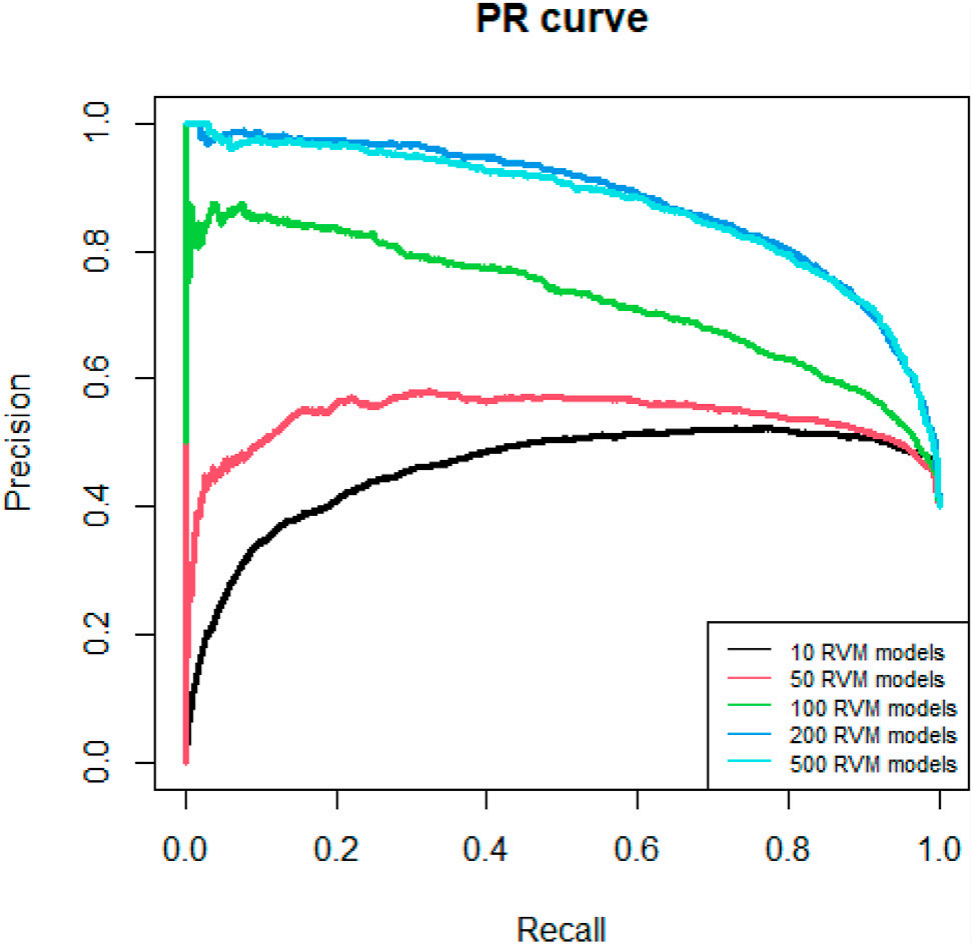
PR curves of different RVM models.

Since AdaRVM needs to construct multiple RVM models, the number of RVM models determines the accuracy of AdaRVM. Although in theory, the more the number of RVM models, the more accurate AdaRVM. But the huge number of RVM will cause the training time to rise sharply, and after reaching a certain amount, the accuracy of AdaRVM will be stable. Therefore, we need to try to build AdaRVM with multiple numbers of RVM models to know the most suitable number of models. We used 10, 50, 100, 200, 500 RVM models to construct AdaRVM respectively. We used 90% data to train the model and used the rest 10% data to test the performance of different AdaRVMs. The AUC and AUPR of these models are shown in Figures 2, 3.

As we can see in Figures 2, 3, as the number of RVM models increases, the AUC and AUPR of AdaRVM have improved significantly. But as the number of RVM reached 200, both AUC and AUPR stabilized and remained at 0.91 and 0.88. Therefore, we used 200 RVM models to construct the final AdaRVM.

After obtaining the final AdaRVM, we need to test the performance of it. 10-cross validation has been applied to test the stability of AdaRVM.

**FIGURE 4:**
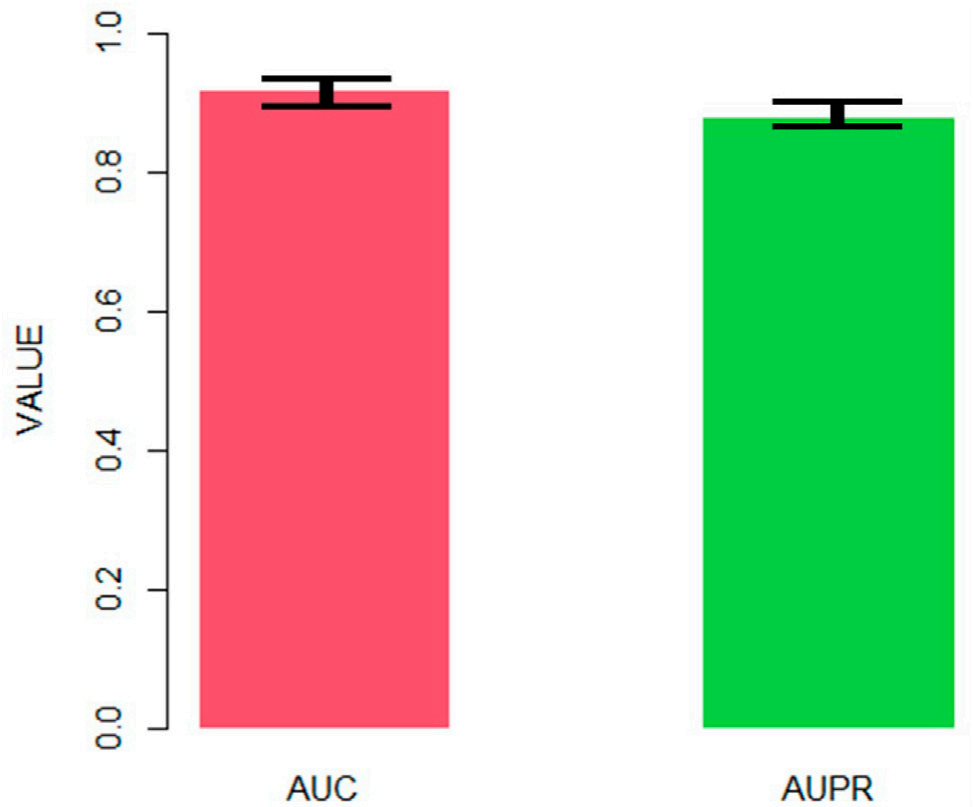
The AUC and AUPR of AdaRVM in 10-cross validation.

Figure 4 shows the AUC and AUPR of AdaRVM in 10-cross validation. Since we tested AdaRVM 10 times, the mean AUC is 0.921 and the mean AUPR is 0.882. The standard deviation is 0.006 and 0.005, respectively.

The experiments showed the high accuracy of AdaRVM. At the same time, in 10 cross-validation experiments, AdaRVM has a small variance of AUC and AUPR, indicating that the performance of the method is very stable, and it is a reliable method for predicting pulmonary injury-related genes.

### Revealing the Functions of Cavin-2

AdaRVM found that Cavin-2 has strong potential to be related to pulmonary injury. To verify our method and explore the function of cavin-2, we used Summary data level Mendelian Randomization (SMR) to integrate GWAS, eQTL, and mQTL to deeply reveal the role of cavin-2 in lung injury.

Lung function indicators contain the lung volume, the forced vital capacity (FVC), the forced expiratory volume in one second (FEV1), and the ratio of FEV1 to FVC (FEV1/FVC), and the peak expiratory flow (PEF). Lung volume represents the volume of gas passing through the lungs at different stages of the respiratory cycle. FVC refers to the maximum amount of air that can be exhaled as soon as possible after inhaling as much as possible. FEV1 is the volume of expiratory breath in the first second after the maximum inhalation. FEV1/FVC is a common indicator to judge airway obstruction and can reflect the type and degree of ventilation disorders. PEF is the maximum flow when exhaling forcefully. These indicators are important basis for the diagnosis of lung diseases. Additionally, when lung damage is caused by a variety of reasons, the interstitium secretes collagen for repair. The excessive repair can lead to the formation of cystic pulmonary fibrosis. Based on the above, we selected GWAS data related to the six lung function injury traits (lung volume, FVC, FEV1, FEV1/FVC, PEF, and CF) for the following analysis.

Four datasets of GWAS data were derived from the same study results (Shrine et al., 2019). These four data individually described FVC, FEV1, FEV1/FVC, and PEF traits. The acquisition of CF GWAS data depends on the BioBank Japan (BBJ) Project, a prospective BioBank that collected different kinds of biological samples from 12 medical institutions in Japan (Sakaue et al., 2021).

To focus the SNPs localized in the region of Cavin-2 gene, we searched the location of Cavin-2 gene in GeneCards website. The site contains detailed information about all type of genes such as protein-coding genes, RNA genes, gene clusters. The gene information showed in this website includes gene aliases, related position information in the genome, gene function, gene mapping, gene involved in pathways, and so on. According to the search results, Cavin-2 gene was located at 192,699,028- 192,711,981 on chromosome 2. Thus, we eliminated SNPs that did not exist in that range. Finally, six GWAS subsets of Cavin-2 gene SNPs were generated. Then we utilized these subsets for the next step of association analysis with eQTL and mQTL.

In our study, we employed four eQTL data including three blood eQTL and one lung tissue eQTL, and one mQTL data. Two datasets of eQTL were downloaded from Genotype-Tissue Expression (GTEx) website. The GTEx project collected and analyzed samples from 53 non-diseased tissues across nearly 1,000 individuals to investigate tissue-specific gene expression patterns. Since we studied lung function traits, we selected one lung eQTL data, and one blood eQTL data. The remaining two eQTL datasets were obtained by RNA sequencing of the whole peripheral blood of 2,116 healthy adults from four Dutch cohort (CODAM, LLD, LLS, and RS). One eQTL data contains 23,060 gene level effects, while the other dataset contain 21,888 exon level effects (Zhernakova et al., 2017). Similarly, the mQTL dataset was downloaded from the same browser. This study analyzed the data from the five Dutch biobank study (CODAM, LLD, LLS, NTR, and RS) of 3,841 whole blood samples (Bonder et al., 2017). The mQTL dataset contained 272,037 independent cis-mQTL effects. The whole datasets used in this paper are listed in Table 1.

**TABLE 1:** The new dataset list for SMR analysis. Thirty SMR datasets were created for this analysis.

GWAS	eQTL	SMR	GWAS	eQTL	SMR
FEV_1_	GTEx_Blood	FEV_1__GTEx_Blood	FVC	GTEx_Blood	FVC_GTEx_Blood
	GTEx_Lung	FEV_1__GTEx_Lung		GTEx_Lung	FVC_GTEx_Lung
	GENE_Blood	FEV_1__GENE_Blood		GENE_Blood	FVC_GENE_Blood
	EXON_Blood	FEV_1__EXON_Blood		EXON_Blood	FVC_EXON_Blood
FEV_1_/FVC	GTEx_Blood	FEV_1_/FVC_GTEx_Blood	PEF	GTEx_Blood	PEF_GTEx_Blood
	GTEx_Lung	FEV_1_/FVC_GTEx_Lung		GTEx_Lung	PEF_GTEx_Lung
	GENE_Blood	FEV_1_/FVC_GENE_Blood		GENE_Blood	PEF_GENE_Blood
	EXON_Blood	FEV_1_/FVC_EXON_Blood		EXON_Blood	PEF_EXON_Blood
Lung volume	GTEx_Blood	LungVolume_GTEx_Blood	CF	GTEx_Blood	CF_GTEx_Blood
	GTEx_Lung	LungVolume_GTEx_Lung		GTEx_Lung	CF_GTEx_Lung
	GENE_Blood	LungVolume_GENE_Blood		GENE_Blood	CF_GENE_Blood
	EXON_Blood	LungVolume_EXON_Blood		EXON_Blood	CF_EXON_Blood

**FIGURE 5:**
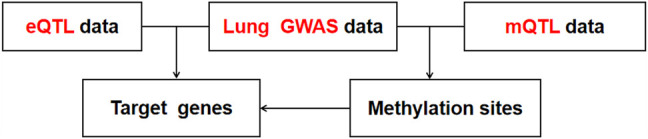
The process of GWAS, eQTL, and mQTL conjoint analysis.

First, we conducted co-localization analysis on two datasets of GWAS and eQTL to find overlapping SNPs and generate a new data containing these SNPs (Figure 5). Then the Z-Score method was used to standardized GWAS and eQTL data for the subsequent analysis. Accoding to the Z-Score formula (① c=qnorm(1−p÷2); Beta>0, c=Z Score, Beta<0;  ②  c=−Z Score; Z Score=Beta÷SE ), the Z value could calculated through using any two of the three values. Beta/OR value refers to the value of regression coefficient, while SE value refers to the standard error of the regression coefficient. Z values could compute the T_SMR_ value using Eq. 16, and then calculated the *p* values (P_SMR_) corresponding to each variation by chi-square test.

$$T_{SMR}=\frac{Z_{GWAS}^{2}*Z_{eQTL}^{2}}{Z_{GWAS}^{2}+Z_{eQTL}^{2}}$$ (16)

P_SMR_ value of 0.05 was taken as the statistically significant threshold. If the P_SMR_ value of some variations is less than 0.05, these variations may affect the pulmonary phenotype by affecting Cavin-2 gene expression. To determine the relationship between these variation points and the classical signal pathways related to lung injury, we performed further signal pathway analysis to verify whether the changes of Cavin-2 gene expression mediated by these variation points could interact with the components of ERK1/2, AKT, and STAT3 classical signal pathways related to lung injury, revealing the function of Cavin-2 gene in lung injury and further confirming the accuracy of our analysis results.

We first conducted the conjoint analysis between the pair of GWAS and eQTL data, and a total of 24 times SMR analyses were performed. First of all, we need to find the same mutation locus between GWAS dataset and eQTL dataset and then combine the information of the mutation locus in the two datasets to generate a new SMR dataset. As the GWAS data subset had a very small list of SNPs, the overlapping SNPs found were much rarer in the new SMR datasets. No more than 10 SNPs were found in all SMR datasets. There were 3 overlapping SNPs in the dataset, which was the dataset containing the most overlapping SNPs. These data sets generally contain only one overlapping susceptible site information.

In total, we discovered 3 susceptible loci in 24 SMR datasets. These SNPs were rs111946466, rs1128965, and rs6718527. These three sites were repeated in these SMR datasets. Half of the SMR datasets contained the rs1128965 information, suggesting this SNPs may closely associated with the Cavin-2 gene expression. Additionally, the rs6718527 site may exhibit a tissue-specific as it is identified only by three SMR datasets containing eQTL information for lung.

Then, we performed SMR analysis on these datasets to verify whether these three loci affect lung functional phenotypes by influencing Cavin-2 gene expression. The Z value of GWAS and eQTL in the dataset was used to calculate the T_SMR_ value of the mutation locus. Then, the Chi-square test of T_SMR_ was performed to obtain the P_SMR_ value. The *p* value was compared with the threshold value of 0.05. It was found that the four data sets had the susceptible site rs1128965. Besides, the P_SMR_ value obtained after analyzing the susceptible site was below 0.05. These four data sets are consistent with the lung function impairment phenotype of FVC and PEF. This suggests that the mutation locus can down-regulate the expression of the Cavin-2 gene, resulting in abnormal lung function of FVC and PEF.

The SMR dataset of the 6 mQTL also had SNPs information of three susceptible loci. The three SNPs include rs12477095, rs11681727, and rs58245883. These three susceptibility loci appeared in all six datasets. However, only the SMR dataset of FEV_1_ trait identified rs12477095 as significant through SMR analysis. This suggests that this site may perform DNA methylation of Cavin-2, affecting the FEV_1_ phenotype associated with lung function.

The Cavin-2 signal pathway was analyzed to further explain the association between the changes in gene expression mediated by Cavin-2-point mutation and ERK1/2, PI3K-AKT signal pathway, and STAT3. The extracellular signal-regulated kinase (ERK) pathway is activated by various extracellular factors, such as growth factors and hormones, to participate in cell proliferation and differentiation, stress response, etc. SDPR is a metastasis inhibitor that inhibits epithelial-mesenchymal transformation (EMT) and migration, promoting apoptosis by interacting with ERK analysis to inhibit the extracellular ERK pathway in breast tumors. Therefore, SDPR may inhibit the ERK pathway to regulate lung injury.

Additionally, AKT-related signaling pathways help regulate cell functions such as cell survival, proliferation, differentiation, and migration. They are also important compensation mechanisms for the body in responding to various harmful stimuli. The PI3K/AKT/eNOS signaling pathway is a signal transduction pathway that plays an essential regulatory role in endothelial cells. The endothelial nitric oxide synthase (eNOS) is an important downstream target of AKT and helps in regulating vascular growth and endothelial function. Cavin-2 regulates nitric oxide (NO) production in endothelial cells by controlling eNOS stability and activity. Cavin-2 can also help regulate lung injury by regulating eNOS associated with the PI3K-Akt signal pathway of lung injury.

As a stress-induced inflammatory signaling pathway, signal transducers and activators of transcription 3 (STAT3) play an essential role in regulating various biological behaviors of a normal organism, such as immune response, tissue repair, cell growth, etc. STAT3 is an indispensable key molecule in the tumorigenesis and tumor-associated inflammation process, which is promoted by chronic inflammation and abnormal activation of STAT3. This causes various diseases since signal transduction and STAT3 help protect respiratory epithelial cells during injury. Cavin-2 is abundant in lung epithelial cells and is closely associated with cell signal transduction. Cavin-2 helps regulate the function of the lung during an injury by affecting signal transduction in the STAT3 signaling pathway.

## Conclusion

Gene mutations play an important role in the occurrence of pulmonary injury, and many mutated genes and genomes have been identified. With the widespread application of machine learning methods in the biomedical field, a large number of researchers use such methods to study disease-related biomolecules on a large scale. But no method has been developed specifically to study pulmonary injury-related genes. In this paper, we developed AdaRVM which used gene expression and gene interaction features to identify pulmonary injury-related genes. We extracted the expression of genes in 13 tissues and 1,212 genes interaction. Due to the high dimension of features, PCA was applied to reduce the dimension and remain the key information. Then, we have determined the method of constructing AdaRVM through 200 RVM models through many experimental attempts. Through the idea of Adaboost, the output results of 200 RVM models are aggregated to obtain the final prediction result.

The 10-cross validation showed the high precision of AdaRVM, with AUC of 0.921 and AUPR of 0.882, which proves the effectiveness of our method. To verify the accuracy of our results, we selected Cavin-2 gene which is predicted as pulmonary injury-related genes by AdaRVM to do case study. We investigated the relationship between the Cavin-2 gene and three signaling pathways associated with lung injury. ERK is a classic pathway of the mitogen-activated protein kinase (MAPK) signal transduction system. Typically, ERK is located in the cytoplasm and is activated only after phosphorylation. ERK regulates the activity of some transcription factors, such as STATs, Jun, Fos, and ATF2, through phosphorylation. These transcription factors further regulate the transcription of their respective target genes, leading to changes in the expression or activity of specific proteins, ultimately regulating cell function, and metabolism. ERK also regulates various activities such as cell growth, apoptosis, and embryogenesis. The ERK1/2 signaling pathway is essential in the EMT process and helps regulate cancer metastasis (Sheng et al., 2017). Typically, EMT occurs in epithelial cells, and various signals can induce it. Members of the transforming growth factor β family (TGFβ) are some important cytokines that can induce the transformation process of EMT. Besides, they can treat fibrotic diseases and tumor diseases. TGFβ signaling can induce EMT conversion through several different signaling mechanisms. The EMT induced by the transforming growth factor (TGF-β1) is important pathogenesis of silicosis fibrosis. TGF-β1 can induce EMT by activating the downstream ERK1/2 pathway and promoting silicosis fibrosis development. SDPR can act as a metastasis inhibitor and regulate the ERK pathway to inhibit EMT. Therefore, SDPR may inhibit the ERK pathway to regulate lung injury. The eNOS is a common downstream substrate of AKT, which is activated to promote eNOS protein expression. The eNOS is a kind of nitric oxide synthase isoenzyme, primarily distributed in vascular endothelium and catalyzes l-arginine to produce NO, which vasodilates and increases vascular permeability.

PI3K/AKT/eNOS is an important signal transduction pathway in endothelial cells. This pathway regulates inflammatory responses and the expression of inflammatory mediators, such as eNOS, primarily through downstream proteins of AKT (Everaert et al., 2010). Inactivation of eNOS is associated with oxidative stress, inflammatory response, and adhesion to the vascular endothelium. Endothelial dysfunction helps in atherosclerosis and ischemia-reperfusion injury. Endothelial dysfunction is characterized by reduced eNOS-induced NO bioavailability, which dilates blood vessels and inhibits inflammation. Cavin-2 also regulates NO production in endothelial cells by controlling the stability and activity of eNOS, and cavin-2 knockdown cells produce much less NO than WT cells. The binding of estrogen, vascular endothelial growth factor, insulin, and other signal molecules with corresponding receptors on the cell surface can activate intracellular PI3K. Besides, the activated PI3K can generate PIP3 through phosphorylation. PIP3 binds to intracellular signals to promote PDK1 phosphorylation of AKT, activating the expression of its downstream target protein.

JAK-STAT comprises three parts: The receptor tyrosine kinase-associated receptor for receiving the signal, the tyrosine kinase JAK for transmitting the signal, and the transcription factor STAT for producing the effect (Johnson et al., 2018). Cytokines or other extracellular signal ligands on the cell membrane induce the aggregation of the corresponding receptors to form homologous or heterologous dimers. This brings the protein JAK together in the cytoplasm, leading to autophosphorylation. Activation of JAK leads to further phosphorylation of the tyrosine residues in the cytoplasmic region of the receptor and mediates STAT3 to bind to the phosphorylated tyrosine residues on the receptor through its Src homology 2 (SH2) domain, phosphorylating the tyrosine residues of STAT3. After phosphorylation, STAT3 is transferred to the nucleus in the form of a homologous or heterologous dimer and binds to the DNA promoter of the target gene to activate the transcription of the target gene. Interstitial lung disease (ILD) includes fibrotic lung diseases characterized by cell proliferation, interstitial inflammation, and fibrosis. The JAK/STAT pathway is activated in response to the interaction of many pro-fibrotic/pro-inflammatory cytokines such as IL-6, IL-11, and IL-13, elevated in various ILD. Similarly, several overexpressed growth factors in ILD, such as platelet-derived growth factor (PDGF), TGF-β1, and fibroblast growth factor (FGF), activate JAK/STAT via classical or non-classical signaling pathways. This suggests that JAK/STAT plays a dominant role in ILD.

## Data Availability

The original contributions presented in the study are included in the article/Supplementary Material, further inquiries can be directed to the corresponding author.
